# Antagonizing STK25 Signaling Suppresses the Development of Hepatocellular Carcinoma Through Targeting Metabolic, Inflammatory, and Pro-Oncogenic Pathways

**DOI:** 10.1016/j.jcmgh.2021.09.018

**Published:** 2021-10-06

**Authors:** Yeshwant Kurhe, Mara Caputo, Emmelie Cansby, Ying Xia, Sima Kumari, Sumit Kumar Anand, Brian W. Howell, Hanns-Ulrich Marschall, Margit Mahlapuu

**Affiliations:** 1 Department of Chemistry and Molecular Biology, University of Gothenburg and Sahlgrenska University Hospital, Gothenburg, Sweden; 2 Department of Neuroscience and Physiology, State University of New York Upstate Medical University, Syracuse, New York; 3 Department of Molecular and Clinical Medicine, Wallenberg Laboratory, Institute of Medicine, University of Gothenburg and Sahlgrenska University Hospital, Gothenburg, Sweden

**Keywords:** STK25, Hepatocellular Carcinoma, NASH, CDAA, DEN, AFP, α-fetoprotein, BrdU, bromodeoxyuridine, CASP3, caspase 3, CDAA, choline-deficient L-amino-acid–defined, DEN, diethylnitrosamine, EMT, epithelial-mesenchymal transition, EpCAM, epithelial cell adhesion molecule, ER, endoplasmic reticulum, ERK, extracellular signal-regulated kinase, GRP78, glucose regulatory protein 78, HCC, hepatocellular carcinoma, JNK, Jun N-terminal kinase, MST, mammalian sterile 20-like, NAFLD, non-alcoholic fatty liver disease, NASH, nonalcoholic steatohepatitis, PCNA, proliferating cell nuclear antigen, ROS, reactive oxygen species, siRNA, small interfering RNA, STAT3, signal transducer and activator of transcription 3, STK25, serine/threonine kinase 25, YAP, Yes-associated protein

## Abstract

**Background & Aims:**

Hepatocellular carcinoma (HCC) is one of the most fatal and fastest-growing cancers. Recently, nonalcoholic steatohepatitis (NASH) has been recognized as a major catalyst for HCC. Thus, additional research is critically needed to identify mechanisms involved in NASH-induced hepatocarcinogenesis, to advance the prevention and treatment of NASH-driven HCC. Because the sterile 20-type kinase serine/threonine kinase 25 (STK25) exacerbates NASH-related phenotypes, we investigated its role in HCC development and aggravation in this study.

**Methods:**

Hepatocarcinogenesis was induced in the context of NASH in *Stk25* knockout and wild-type mice by combining chemical procarcinogens and a dietary challenge. In the first cohort, a single injection of diethylnitrosamine was combined with a high-fat diet-feeding. In the second cohort, chronic administration of carbon tetrachloride was combined with a choline-deficient L-amino-acid–defined diet. To study the cell-autonomous mode of action of STK25, we silenced this target in the human hepatocarcinoma cell line HepG2 by small interfering RNA.

**Results:**

In both mouse models of NASH-driven HCC, the livers from *Stk25*^-/-^ mice showed a markedly lower tumor burden compared with wild-type controls. We also found that genetic depletion of STK25 in mice suppressed liver tumor growth through reduced hepatocellular apoptosis and decreased compensatory proliferation, by a mechanism that involves protection against hepatic lipotoxicity and inactivation of STAT3, ERK1/2, and p38 signaling. Consistently, silencing of STK25 suppressed proliferation, apoptosis, migration, and invasion in HepG2 cells, which was accompanied by lower expression of the markers of epithelial–mesenchymal transition and autophagic flux.

**Conclusions:**

This study provides evidence that antagonizing STK25 signaling hinders the development of NASH-related HCC and provides an impetus for further analysis of STK25 as a therapeutic target for NASH-induced HCC treatment in human beings.

## Graphical abstract

**Figure:**
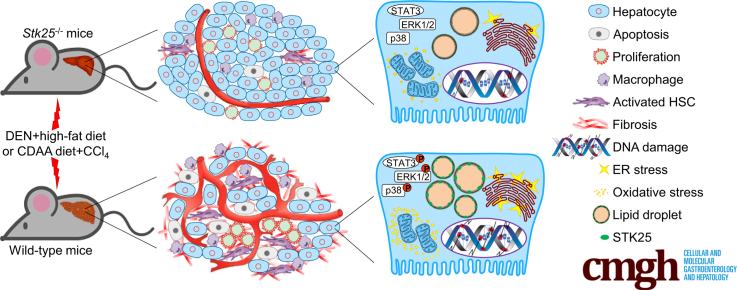

**Summary**

We provide evidence that antagonizing STK25 activity hinders the development of hepatocellular carcinoma, which is one of the most fatal cancers. This study provides an impetus for further analysis of STK25 as a therapeutic target in liver cancer.

Worldwide, liver cancer is the sixth most common cancer and the second leading cause of cancer-related deaths (approximately 800,000 per year, a figure that is increasing).^1^ Hepatocellular carcinoma (HCC) constitutes approximately 90% of primary liver cancers and is refractory to nearly all currently available anticancer therapies.^1^ The well-known risk factors for HCC are hepatitis B and C virus infections and alcohol abuse. Recently, attention has focused on the importance of nonalcoholic steatohepatitis (NASH) in the development of HCC. First report linking NASH to HCC appeared in 2001,^2^ and, to date, NASH has been reported in several studies as the single most common and rapidly increasing underlying etiology of HCC.^3^, ^4^, ^5^

NASH is a severe stage of nonalcoholic fatty liver disease (NAFLD) and is characterized by hepatic lipid accumulation (ie, steatosis), meta-inflammation, and cellular damage (hallmarked by hepatocyte ballooning) with different stages of local fibrosis.^6^^,^^7^ The prevalence of NASH is increasing rapidly because of the pandemic spread of obesity, which is the main risk factor of NAFLD/NASH.^4^ The HCC incidence among NASH patients can vary from 2.4% to 12.8%^8^; however, the nature of the signals that underpin the aggravation of NASH into HCC remains elusive and it is not known why some NASH patients rapidly progress to HCC while others do not. Importantly, compared with HCC triggered by viral infection or alcohol, NASH-related HCC is reportedly less likely to be detected at surveillance and more likely to be treated less effectively.^9^^,^^10^ Thus, there is a strong medical need for new predictors and improved treatments of NASH-driven HCC.

Our recent translational studies have identified the liver lipid droplet–decorating protein kinase serine/threonine kinase 25 (STK25, also referred to as YSK1 or SOK1) as a new regulator of hepatic lipid partitioning.^11^, ^12^, ^13^, ^14^, ^15^, ^16^, ^17^ Furthermore, we found that STK25 critically controls liver meta-inflammation, oxidative and endoplasmic reticulum (ER) stress, as well as the autophagic degradation system,^12^, ^13^, ^14^, ^15^, ^16^, ^17^ which all have been described as key factors leading to HCC development in the context of NASH.^8^^,^^18^^,^^19^ On the basis of this evidence, we now hypothesize that STK25 is an important mediator in the complex and integrated molecular network driving the initiation and progression of NASH-related HCC, and a potential target for anti-HCC therapy.

To decipher the possible role of STK25 in NASH-driven HCC, we here used the *Stk25*^-/-^ mouse model, in which hepatocarcinogenesis was induced in the context of NASH by combining chemical procarcinogens and a dietary challenge. In the first cohort of *Stk25*^-/-^ mice and wild-type littermates, HCC was triggered by a single injection of diethylnitrosamine (DEN), which is known to alkylate DNA and promote oxidative stress via the production of reactive oxygen species (ROS),^20^^,^^21^ combined with long-term high-fat diet–feeding to induce hepatic steatosis, inflammation, and fibrosis (ie, the spectrum of lesions present in NASH patients). In the second cohort of mice, the choline-deficient L-amino-acid–defined (CDAA) diet was used to produce liver steatosis, meta-inflammation, and nutritional fibrosis,^22^, ^23^, ^24^ and this dietary regimen was combined with chronic low-dose administration of carbon tetrachloride (CCl_4_), a toxin that accelerates extracellular matrix deposition and necrosis in the liver through its metabolite CCl_3_.^25^^,^^26^ Our study identifies STK25 signaling as a critical driver of the initiation and progression of HCC by showing that genetic ablation of STK25 efficiently suppressed tumor development in 2 mouse models of hepatocarcinogenesis that closely recapitulated the nature of human HCC.

## Results

### Genetic Ablation of STK25 Hinders the Development of HCC in Mice

**Figure 1:**
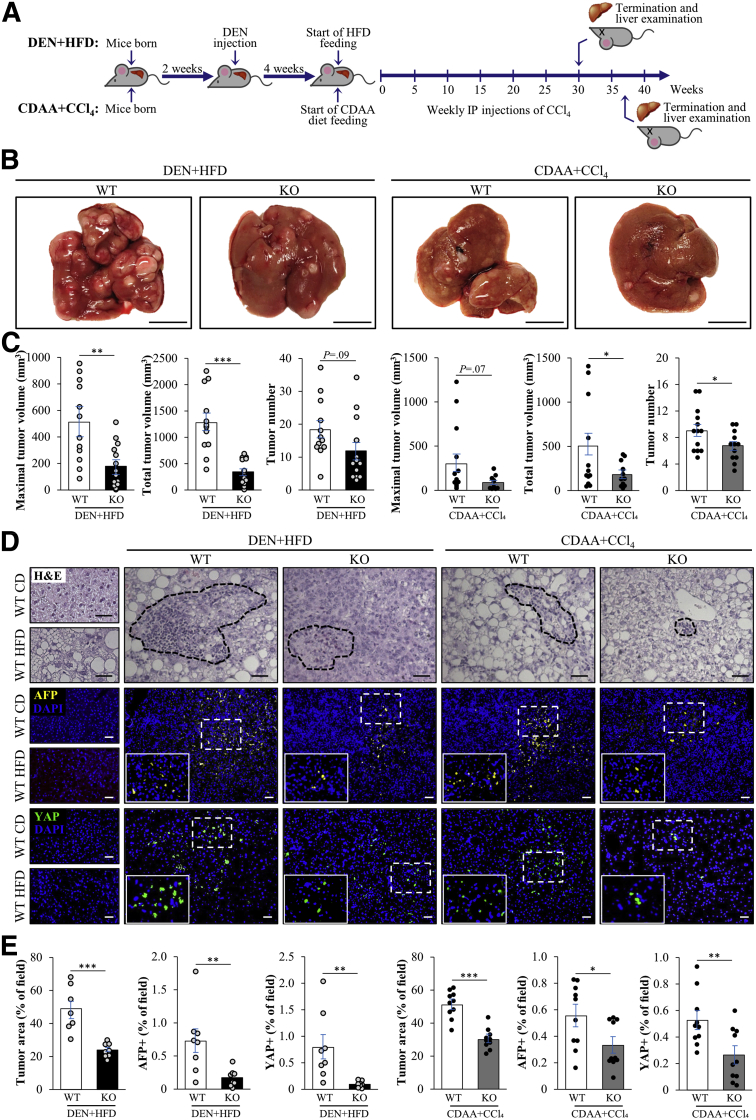
**Genetic ablation of STK25 suppresses tumor development in DEN- or CDAA-induced hepatocarcinogenesis models in mice.** (*A*) Schematic presentation of the experimental design of the in vivo studies. (*B*) Representative images of whole liver. *Scale bars*: 1 cm. (*C*) Quantification of tumor volume and number. (*D*) Representative liver sections stained with H&E or processed for immunofluorescence with anti-AFP (yellow) or anti-YAP (green) antibodies; nuclei stained with DAPI (blue). *Scale bars*: 50 μm. (*E*) Quantification of the tumor area and immunofluorescence staining. Data are means ± SEM from 8 to 14 mice per group. CD, chow diet; DAPI, 4′,6-diamidino-2-phenylindole; HFD, high-fat diet; IP, intraperitoneal; KO, knockout; WT, wild-type. ∗*P* < .05, ∗∗*P* < .01, and ∗∗∗*P* < .001.

**Figure 2:**
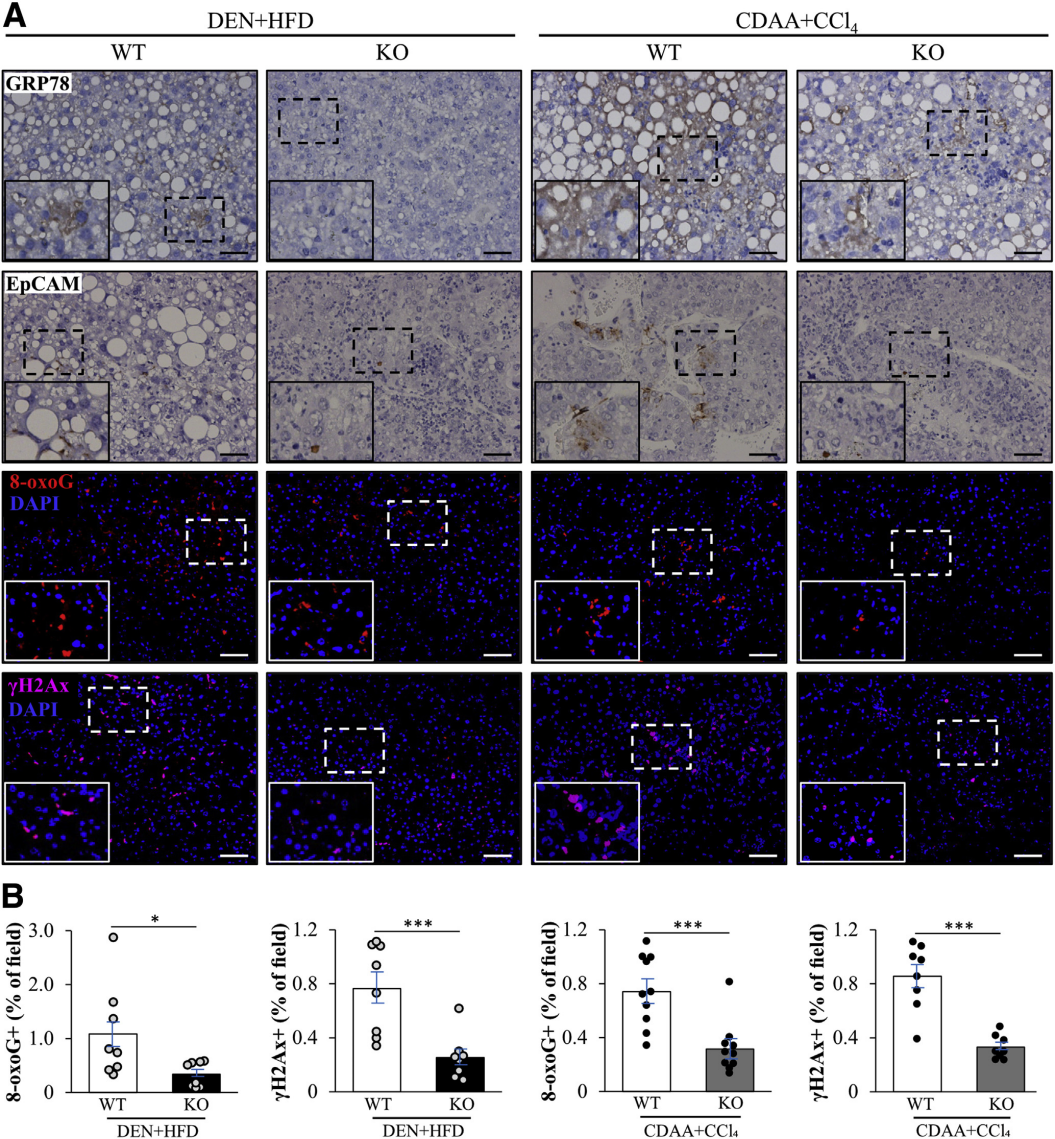
**Knockout of STK25 in mice with DEN- or CDAA-induced HCC suppresses GRP78 and EpCAM abundance and reduces DNA damage.** (*A*) Representative liver sections processed for immunohistochemistry/immunofluorescence with anti-GRP78 (brown), anti-EpCAM (brown), anti–8-oxoguanine (red), or anti-γH2Ax (pink) antibodies; counterstaining with hematoxylin in immunohistochemistry images; nuclei stained with DAPI (blue) in immunofluorescence images. (*B*) Quantification of the immunofluorescence staining. *Scale bars*: 50 μm. Data are means ± SEM from 8 to 10 mice per group. DAPI, 4′,6-diamidino-2-phenylindole; HFD, high-fat diet; KO, knockout; WT, wild-type. ∗*P* < .05, ∗∗∗*P* < .001.

We used the *Stk25*^-/-^ mouse model, which has a germline ablation of STK25,^27^ to test the hypothesis that STK25 contributes to the initiation and aggravation of DEN-induced HCC during overnutrition (Figure 1*A*). We found that all DEN-injected wild-type mice, following high-fat diet–feeding for 30 weeks, developed numerous macroscopic nodules of variable size in the liver (Figure 1*B*). Interestingly, we observed that the maximal and total volume of visible HCC tumors was reduced by 2.9- ± 0.5-fold and 3.7- ± 0.5-fold, respectively, by the loss of STK25 in knockout mice compared with their wild-type littermates (Figure 1*C*). Furthermore, there was a tendency for lower numbers of surface HCC tumors in *Stk25*^-/-^ livers (Figure 1*C*). Together, these results suggest that both tumor initiation and growth were affected by STK25 ablation. Consistent with these morphologic investigations in whole-liver samples, we found less-extensive damage in H&E-stained liver sections from *Stk25*^-/-^ vs wild-type mice, associated with a significantly reduced labeling for HCC markers α-fetoprotein (AFP) and Yes-associated protein (YAP) (Figure 1*D* and *E*).^28^^,^^29^ Furthermore, immunohistologic assessment indicated that tumors from wild-type mice expressed higher levels of glucose regulatory protein 78 (GRP78) and epithelial cell adhesion molecule (EpCAM), whose abundance is known to correlate with HCC grade and poor patient prognosis (Figure 2*A*).^30^

We also compared CDAA diet–induced HCC carcinogenesis in *Stk25*^-/-^ and wild-type mice (Figure 1*A*). We observed that all wild-type mice fed a CDAA diet and injected with a low dose of CCl_4_ for 37 weeks developed numerous visible tumors in the liver (Figure 1*B*). In agreement with the DEN-induced HCC model, the tumor load was reduced in livers from *Stk25*^-/-^ vs wild-type mice, including a decrease in the maximal and total volume as well as the number of macroscopic HCC nodules (Figure 1*C*). Furthermore, we detected a significantly smaller region with cellular damage in H&E-stained liver sections from *Stk25*^-/-^ vs wild-type mice along with reduced immunostaining for AFP, YAP, GRP78, and EpCAM (Figures 1*D* and *E* and 2*A*).

Together, these 2 translational models of HCC highlight STK25 as a critical regulator of hepatocarcinogenesis.

### STK25 Abrogation Alleviates HCC Through Suppressed Hepatocyte Proliferation and Decreased Angiogenesis

**Figure 3:**
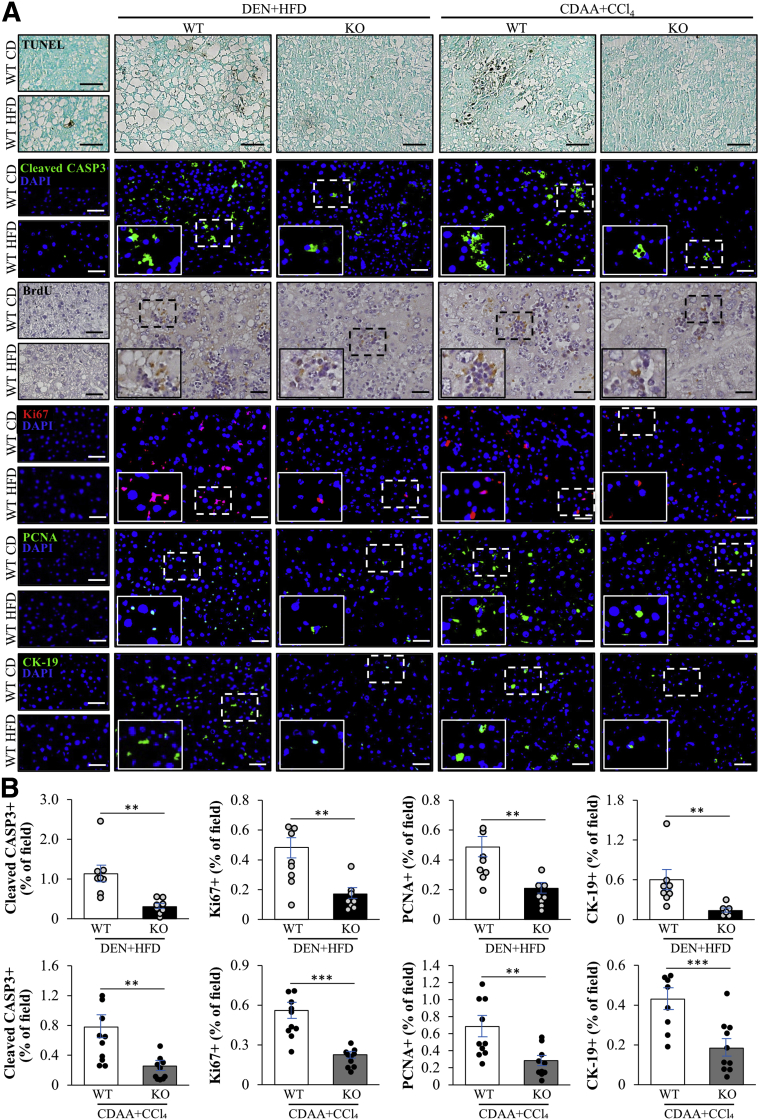
**STK25 deficiency attenuates apoptosis and proliferation in mouse models of DEN- or CDAA-induced HCC.** (*A*) Representative liver sections stained with terminal deoxynucleotidyl transferase–mediated deoxyuridine triphosphate nick-end labeling (TUNEL) or processed for immunohistochemistry/immunofluorescence with anti-cleaved CASP3 (green), anti-BrdU (brown), anti-Ki67 (red), anti-PCNA (green), or anti–cytokeratin-19 (anti–CK-19) (green) antibodies; counterstaining with methyl green (TUNEL) or hematoxylin (BrdU); nuclei stained with DAPI (blue) in immunofluorescence images. (*B*) Quantification of the staining. *Scale bars*: 50 μm. Data are means± SEM from 8 to 10 mice per group. CD, chow diet; DAPI, 4′,6-diamidino-2-phenylindole; HFD, high-fat diet; KO, knockout; WT, wild-type. ∗∗*P* < .01, ∗∗∗*P* < .001.

To explore the mechanisms by which a depletion of STK25 exerts a tumor-mitigating effect, we first examined whether the loss of STK25 decreased the susceptibility of knockout mice to hepatocarcinogenesis by augmenting cell death. On the contrary, we observed significantly reduced apoptosis in the livers from *Stk25*^-/-^ mice compared with wild-type controls, as shown by less terminal deoxynucleotidyl transferase–mediated deoxyuridine triphosphate nick-end labeling (TUNEL)– as well as cleaved caspase 3 (CASP3)-positive cells. This applied both to DEN- and CDAA-induced models of HCC (Figure 3).

It has been shown previously that hepatocyte apoptosis stimulates the compensatory proliferation in liver tissue that in turn promotes carcinogenesis in HCC.^31^, ^32^, ^33^, ^34^ Based on this evidence, we next examined whether STK25 deficiency also suppresses the proliferation of hepatocytes. Indeed, we detected less bromodeoxyuridine (BrdU)-positive cells in the livers from *Stk25*^-/-^ mice than in wild-type mice (Figure 3*A*). Consistently, 3 independent methods of evaluating hepatocyte proliferation—immunostaining for proliferation markers Ki67, proliferating cell nuclear antigen (PCNA), and cytokeratin-19—all showed significantly decreased numbers of proliferating cells in the livers from *Stk25*^-/-^ mice as opposed to wild-type controls (Figure 3), and this difference was observed both in DEN- and CDAA-induced models of HCC.

**Figure 4:**
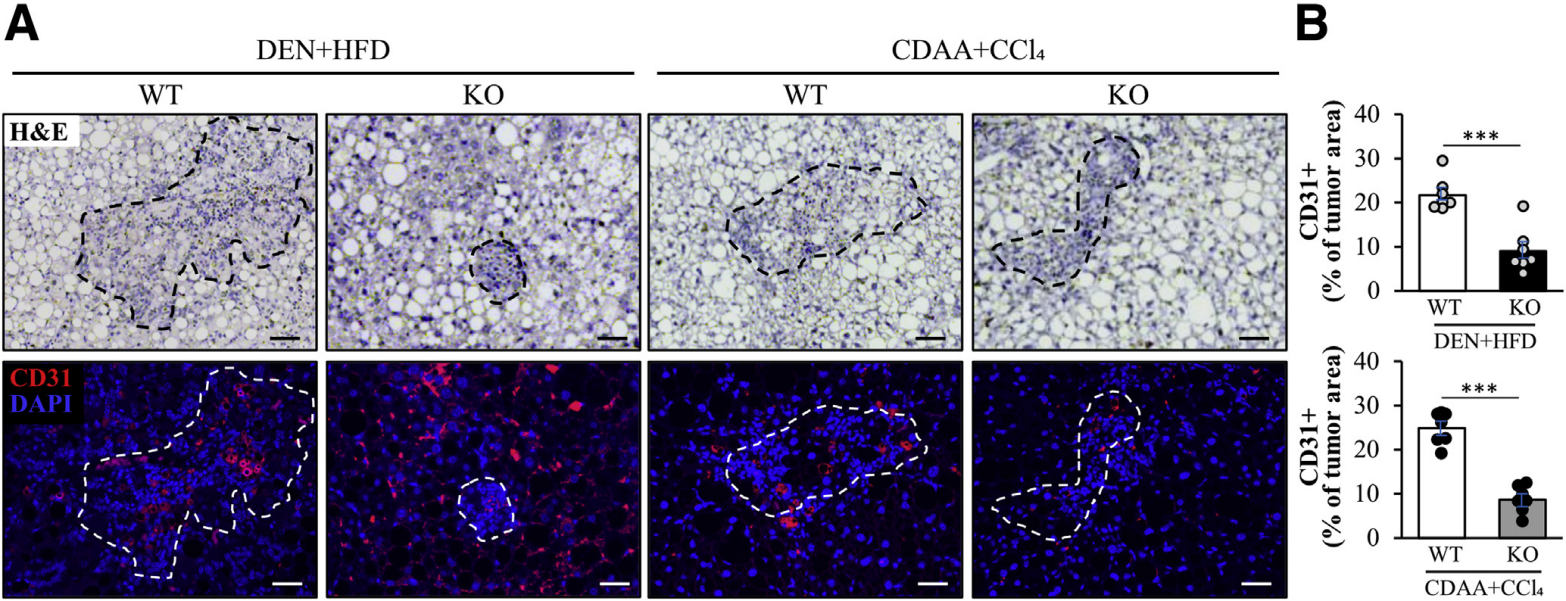
**Knockout of STK25 reduces tumor angiogenesis in mouse models of DEN- or CDAA-induced HCC.** (*A*) Representative liver sections stained with H&E or processed for immunofluorescence with anti-CD31 (red) antibody; nuclei stained with DAPI (blue) in immunofluorescence images. (*B*) Staining with anti-CD31 antibody was selectively quantified in tumor tissue identified from the H&E staining on a serial section (shown by *dashed lines*). *Scale bars*: 50 μm. Data are means ± SEM from 7 mice per group. DAPI, 4′,6-diamidino-2-phenylindole; HFD, high-fat diet; KO, knockout; WT, wild-type. ∗∗∗*P* < .001.

Tumor angiogenesis is an important driver of hepatocellular tumor growth.^35^^,^^36^ We therefore investigated tumor vascularization by immunostaining of liver sections with an anti-CD31 antibody. In both models of HCC, we found a significant reduction in vessel density in tumors from *Stk25* knockout mice compared with their wild-type littermates (Figure 4).

Together, these data suggest that STK25 abrogation confers resistance to hepatocyte apoptosis induced by DEN or CDAA challenge, and suppresses HCC development through attenuated tumor cell proliferation and angiogenesis.

### STK25 Deficiency Protects HCC-Bearing Mice Against Diet-Induced Liver Steatosis, Meta-Inflammation, and Fibrosis

**Figure 5:**
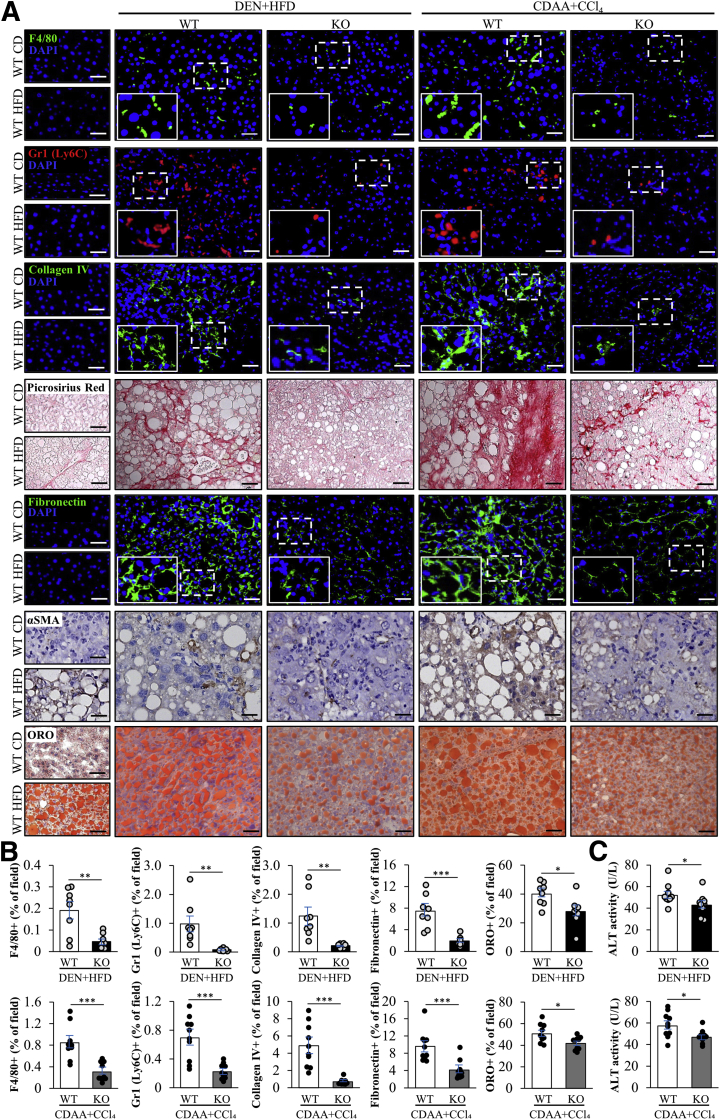
**Genetic ablation of STK25 in mice with DEN- or CDAA-induced HCC suppresses liver steatosis, inflammation, and fibrosis.** (*A*) Representative liver sections stained with Picrosirius Red or Oil Red O, or processed for immunohistochemistry/immunofluorescence with anti-F4/80 (green), anti-Gr1 (Ly6C) (red), anti–collagen IV (green), anti-fibronectin (green), or αSMA (brown) antibodies; nuclei stained with DAPI (blue) in immunofluorescence images; counterstaining with hematoxylin in immunohistochemistry images. (*B*) Quantification of the staining. (*C*) Activity of plasma alanine aminotransferase (ALT). *Scale bars*: 50 μm. Data are means ± SEM from 8 to 10 mice per group. CD, chow diet; DAPI, 4′,6-diamidino-2-phenylindole; HFD, high-fat diet; KO, knockout; ORO, Oil Red O; WT, wild-type. ∗*P* < .05, ∗∗*P* < .01, and ∗∗∗*P* < .001.

Compensatory proliferation after liver damage has been suggested to be triggered by hepatic inflammation mediated primarily by macrophages.^28^^,^^31^^,^^32^ Hepatic macrophages can arise either from circulating bone marrow–derived monocytic precursors or from self-renewing embryo-derived local macrophages, termed *Kupffer cells*^37^; both macrophage populations are positive for F4/80 while freshly infiltrating monocyte-derived macrophages also are characterized by high expression of Gr1 (Ly6C). Here, we found that loss of STK25 protected against hepatic macrophage infiltration in both DEN- and CDAA-induced models of HCC as evidenced by a lower number of F4/80- and Gr1 (Ly6C)-positive cells in the liver sections from *Stk25*^-/-^ vs wild-type mice (Figure 5*A* and *B*). Furthermore, HCC-bearing *Stk25* knockout mice had reduced hepatic fibrosis as shown by reduced staining for collagen IV, Picrosirius Red (stains both collagen type I and type III), and fibronectin (Figure 5*A* and *B*). Consistently, immunostaining for α smooth muscle actin (αSMA), a marker for activated hepatic stellate cells, was lower in liver sections from *Stk25*^-/-^ mice (Figure 5*A*). Diet-induced lipid accumulation in hepatocytes also was mitigated by STK25 abrogation in both models of HCC as shown by a smaller area stained with Oil Red O, which labels neutral lipids (Figure 5*A* and *B*). The improvement in histopathology correlated with lower plasma alanine transaminase (ALT) levels in *Stk25*^-/-^ vs wild-type mice (Figure 5*C*).

Together, in addition to inhibition of liver tumorigenesis, loss of STK25 in both HCC models substantially suppressed all the pathologic features of NASH, including hepatic steatosis, inflammation, and fibrosis.

### Genetic Ablation of STK25 Suppresses Oxidative and ER Stress in the Liver

**Figure 6:**
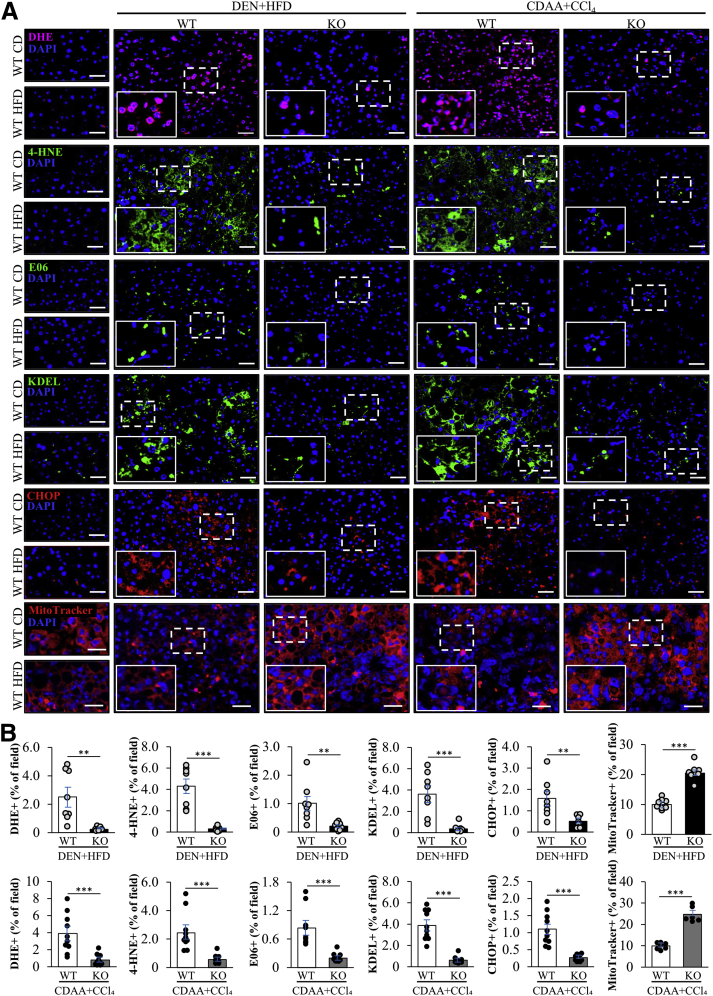
**STK25 disruption in mice protects against hepatic oxidative and ER stress in HCC.** (*A*) Representative liver sections stained with dihydroethidium (DHE) (pink) or MitoTracker Red (red), or processed for immunofluorescence with anti–4-hydroxynonenal (anti–4-HNE) (green), anti-E06 (green), anti-KDEL (green), or anti–C/EBP homologous protein (anti-CHOP) (red) antibodies; nuclei stained with DAPI (blue). (*B*) Quantification of the staining. *Scale bars*: 50 μm; except for MitoTracker Red staining: *scale bars*: 25 μm. Data are means ± SEM from 6 to 10 mice per group. CD, chow diet; DAPI, 4′,6-diamidino-2-phenylindole; HFD, high-fat diet; KO, knockout; WT, wild-type. ∗∗*P* < .01, ∗∗∗*P* < .001.

ER stress and accumulation of ROS within liver parenchymal cells result in compensatory proliferation of differentiated hepatocytes that are otherwise quiescent, which is one of the major pathogenic mechanisms underlying HCC development.^38^ Indeed, we found that lower hepatocyte proliferation in HCC-bearing *Stk25* knockout mice was accompanied by reduced oxidative stress as evidenced by repressed levels of superoxide radicals (O_2_^•−^) quantified by dihydroethidium staining, abrogated oxidative DNA damage detected by immunostaining for 8-oxoguanine and γH2Ax, and protection against deposition of lipid peroxidation products and oxidized phospholipids measured by immunostaining for 4-hydroxynonenal (4-HNE) and E06, respectively (Figures 2*A* and *B* and 6*A* and *B*). In parallel with these alterations in oxidative stress markers, we detected significantly reduced immunostaining for KDEL (a signal motif for ER retrieval) and C/EBP homologous protein (CHOP; a marker for ER stress–induced cell death) in the livers from *Stk25* knockout mice with both DEN- and CDAA-induced HCC (Figure 6*A* and *B*).

Aberrant mitochondrial activity, leading to heightened ROS production, is known to be associated with liver tumorigenesis.^39^^,^^40^ Consistent with higher oxidative stress, we found that mitochondrial activity was approximately 2-fold lower in the livers from wild-type mice compared with *Stk25*^-/-^ mice (Figure 6*A* and *B*).

### STK25 Disruption Reduces ERK1/2, p38, and STAT3 Signaling in the DEN-Induced HCC Model

**Figure 7:**
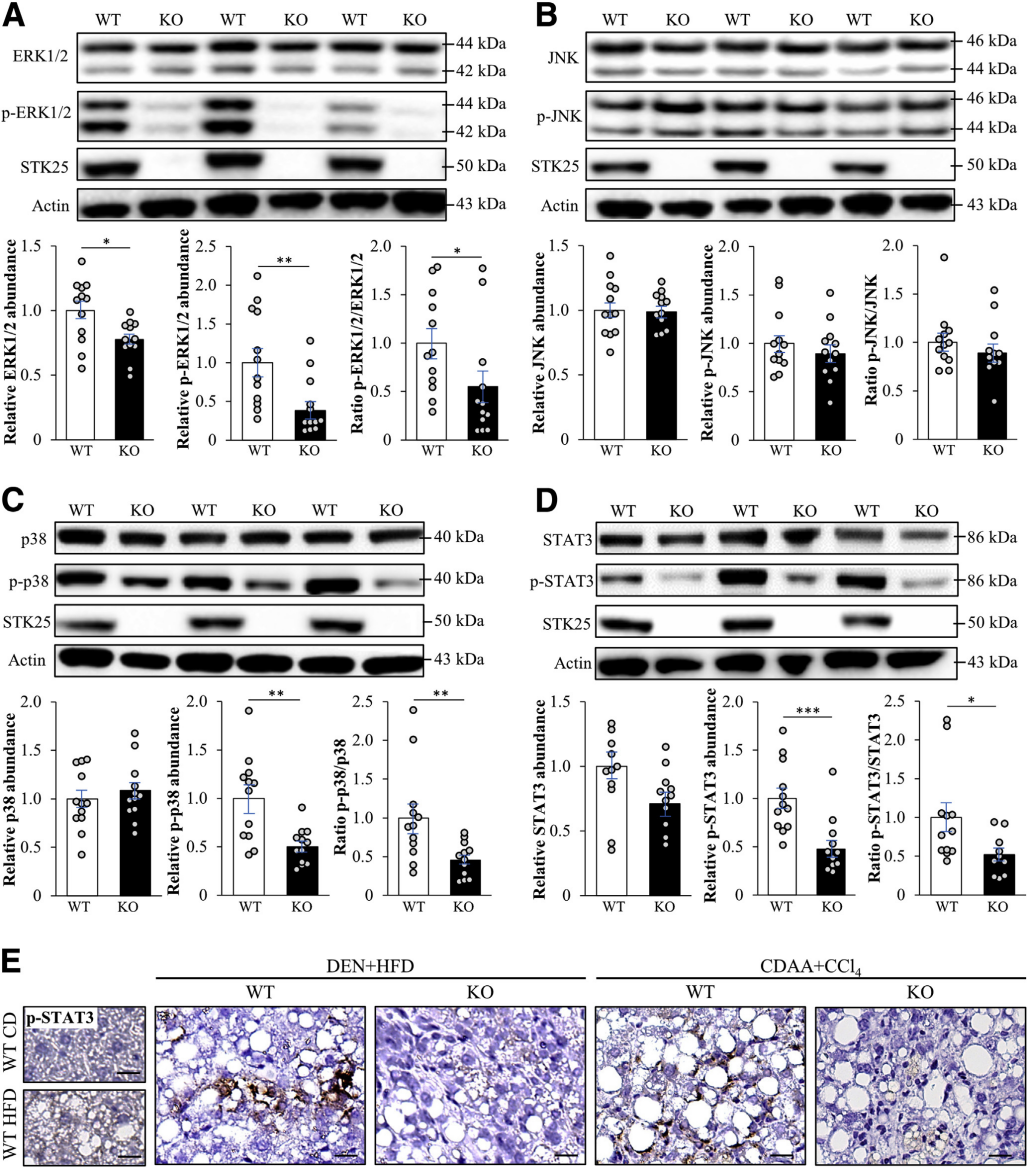
**Genetic ablation of STK25 in a mouse model of DEN-induced HCC reduces the activation of key markers of tumorigenesis.** (*A–D*) Lysates from liver tumors were analyzed by Western blot using antibodies specific for ERK1/2, phospho-ERK1/2 (Thr^202^/Tyr^204^), JNK, phospho-JNK (Thr^183^/Tyr^185^), p38, phospho-p38 (Thr^180^/Tyr^182^), STAT3, phospho-STAT3 (Thr^705^), and STK25. Protein levels were analyzed by densitometry; representative Western blots from 3 different mice per group are shown with actin used as a loading control. (*E*) Representative liver sections processed for immunohistochemistry with anti–phospho-STAT3 antibody; counterstaining with hematoxylin. *Scale bars*: 50 μm. Data are means ± SEM from 12 mice per group. CD, chow diet; HFD, high-fat diet; KO, knockout; WT, wild-type. ∗*P* < .05, ∗∗*P* < .01, and ∗∗∗*P* < .001.

To explore the mechanisms by which STK25 depletion protects against hepatocellular carcinogenesis, we performed immunoblot analysis in the liver tumors isolated from mice with DEN-induced HCC to monitor the hepatic activation status of mitogen-activated protein kinases (MAPKs) extracellular signal-regulated kinase 1/2 (ERK1/2), Jun N-terminal kinase (JNK), and p38, which are important signaling components controlling proliferation, differentiation, and migration in HCC.^41^ We observed higher phosphorylation levels of ERK1/2 as well as p38 MAPK, shown to be activated in the majority of human HCCs,^30^^,^^42^, ^43^, ^44^ in the liver tumors from wild-type vs *Stk25*^-/-^ mice (Figure 7*A* and *C*). Notably, hepatic JNK activation, known to be required for DEN-induced HCC in mice,^45^ was not altered by depletion of STK25 (Figure 7*B*).

It recently was shown that the inactivation of negative regulators of signal transducer and activator of transcription 3 (STAT3) signaling drives the development of HCC in obese mice without affecting NASH or fibrosis.^30^ Consistently, STAT3 activation is enhanced in human HCC specimens.^42^^,^^46^ Given this critical role of STAT3 in HCC progression, we next determined the activation state of STAT3 in liver tumors by Western blot analysis. We found that phosphorylation of STAT3 was significantly lower in tumors from *Stk25* knockout mice when compared with wild-type controls (Figure 7*D*). Reduced STAT3 activation also was observed in liver sections from *Stk25*^-/-^ mice with both DEN- and CDAA-induced HCC by immunohistochemistry analysis using anti–phospho-STAT3 antibody (Figure 7*E*).

Together, these results indicate that STK25 deficiency in hepatocytes protects against tumor development with suppression of several molecular signaling pathways typically activated in aggressive human HCC.

### Silencing of STK25 Inhibits Metastatic Capacity of HepG2 Cells

**Figure 8:**
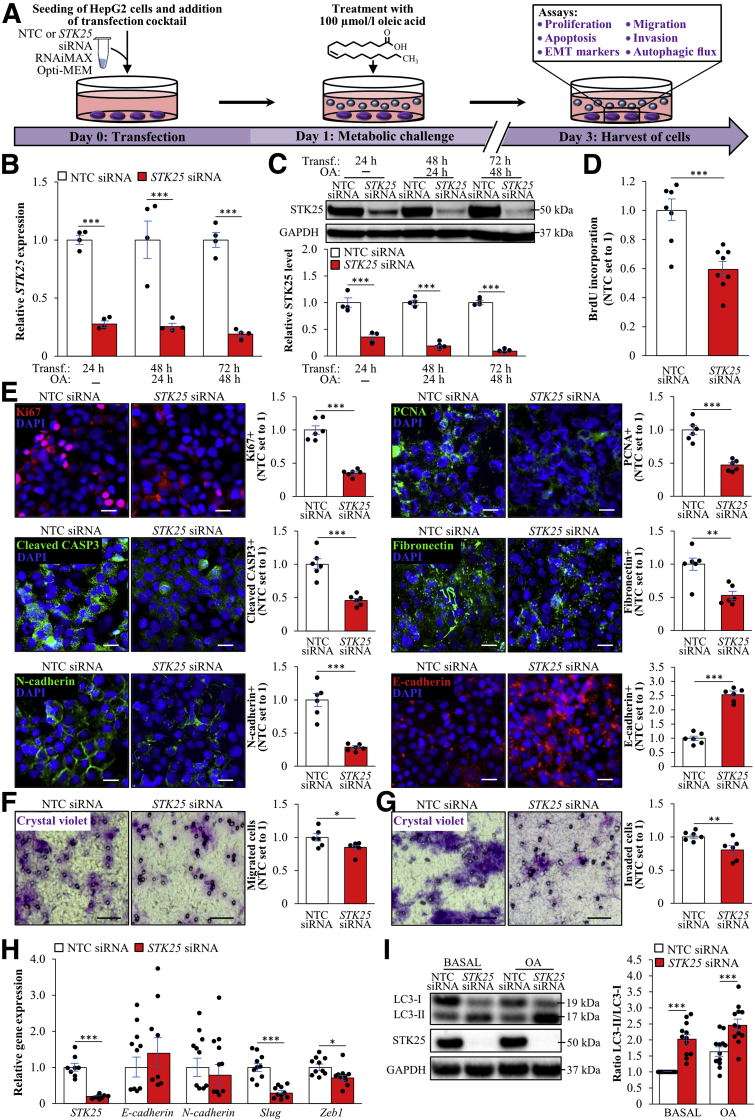
**Silencing of STK25 in human HCC cells treated with oleic acid inhibits proliferation, mobility, and invasiveness.** HepG2 cells were transfected with *STK25* siRNA or nontargeting control (NTC) siRNA and challenged with oleic acid for 48 hours. (*A*) Schematic illustration of the study design. (*B* and *C*) STK25 (*B*) messenger RNA and (*C*) protein abundance. Protein levels were analyzed by densitometry; representative Western blots are shown with glyceraldehyde-3-phosphate dehydrogenase (GAPDH) used as a loading control. (*D*) The proliferation rate was assessed by measuring the DNA synthesis using a Biotrak cell proliferation enzyme-linked immunosorbent assay. (*E*) Representative images of cells processed for immunofluorescence with anti-Ki67 (red), anti-PCNA (green), anti–cleaved CASP3 (green), antifibronectin (green), anti–N-cadherin (green), or anti–E-cadherin (red) antibodies; nuclei stained with DAPI (blue). Quantification of the staining. (*F* and *G*) Representative images of (*F*) migrated and (*G*) invaded cells stained with crystal violet. Quantification of the staining. (*H*) Relative messenger RNA expression of selected EMT markers. (*I*) Cell lysates were analyzed by Western blot using antibodies specific for LC3 and STK25. Protein levels were analyzed by densitometry; representative Western blots are shown with GAPDH used as a loading control. (*E*) *Scale bars*: 15 μm; (*F* and *G*) *scale bars*: 50 μm. Data are means ± SEM from 4 to 10 wells per group. DAPI, 4′,6-diamidino-2-phenylindole; OA, oleic acid; Transf, transfection. ∗*P* < .05, ∗∗*P* < .01, and ∗∗∗*P* < .001.

**Figure 9:**
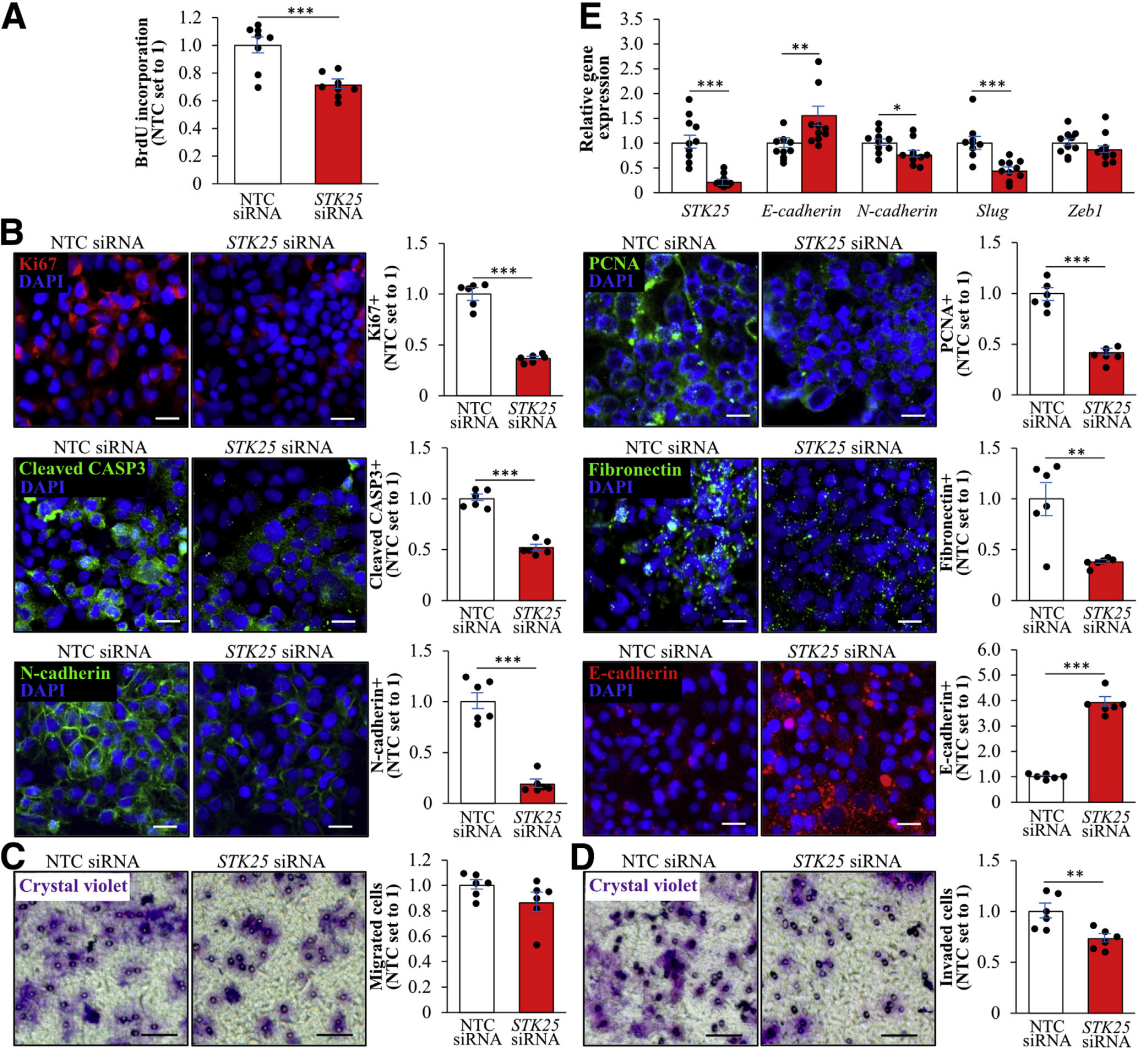
**Silencing of STK25 in human HCC cells maintained under basal culture conditions inhibits proliferation, mobility, and invasiveness.** HepG2 cells were transfected with *STK25* siRNA or nontargeting control (NTC) siRNA. (*A*) The proliferation rate was assessed by measuring the DNA synthesis using a Biotrak cell proliferation enzyme-linked immunosorbent assay. (*B*) Representative images of cells processed for immunofluorescence with anti-Ki67 (red), anti-PCNA (green), anti–cleaved CASP3 (green), anti-fibronectin (green), anti–N-cadherin (green), or anti–E-cadherin (red) antibodies; nuclei stained with DAPI (blue). Quantification of the staining. (*C* and *D*) Representative images of (*C*) migrated and (*D*) invaded cells stained with crystal violet. Quantification of the staining. (*E*) Relative messenger RNA expression of selected EMT markers. (*B*) *Scale bars*: 15 μm; (*C* and *D*), *scale bars*: 50 μm. Data are means ± SEM from 6 to 10 wells per group. DAPI, 4′,6-diamidino-2-phenylindole. ∗*P* < .05, ∗∗*P* < .01, and ∗∗∗*P* < .001.

**Figure 10:**
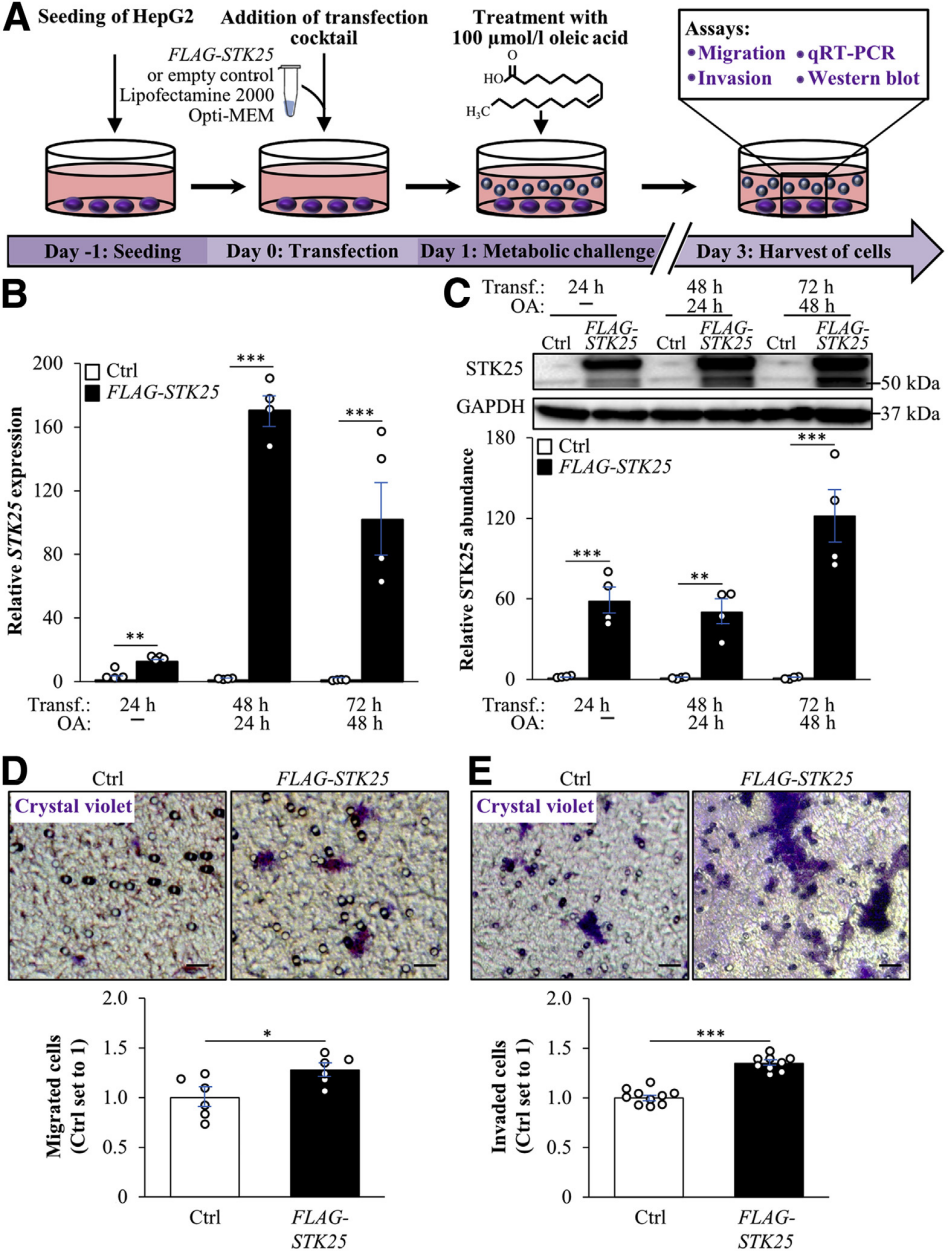
**Overexpression of STK25 in human HCC cells increases mobility and invasiveness.** HepG2 cells were transfected with *FLAG-STK25* or empty control plasmid and challenged with oleic acid for 48 hours. (*A*) Schematic illustration of the study design. (*B* and *C*) STK25 (*B*) messenger RNA and (*C*) protein abundance. Protein levels were analyzed by densitometry; representative Western blots are shown with glyceraldehyde-3-phosphate dehydrogenase (GAPDH) used as a loading control. (*D* and *E*) Representative images of (*D*) migrated and (*E*) invaded cells stained with crystal violet. Quantification of the staining. (*D* and *E*) *Scale bars*: 25 μm. Data are means ± SEM from 4 to 10 wells per group. Ctrl, control; OA, oleic acid; qRT-PCR, quantitative reverse-transcription polymerase chain reaction; Transf., transfection. ∗*P* < .05, ∗∗*P* < .01, and ∗∗∗*P* < .001.

To further study the cell-autonomous mechanisms behind the tumor-mitigating effects of STK25 knockdown, we silenced this target in HepG2 hepatocellular carcinoma cells by a small interfering RNA (siRNA) approach. To mimic conditions in high-fat diet–fed mice and high-risk subjects, we subsequently exposed the cells for 48 hours to 100 μmol/L oleic acid, known to efficiently induce steatosis in vitro (Figure 8*A–C*). Consistent with in vivo studies, STK25 knockdown in HepG2 cells significantly suppressed proliferation as shown by measuring the DNA synthesis rate as well as by staining for the proliferation markers Ki67 and PCNA (Figure 8*D* and *E*). We also found reduced apoptosis in STK25-deficient HepG2 cells as indicated by a decreased amount of cleaved CASP3-positive cells (Figure 8*E*). Migration/invasive capacity, quantified using a Transwell assay, also was suppressed significantly in HepG2 cells transfected with *STK25* siRNA compared with nontargeting control siRNA (Figure 8*F* and *G*). Notably, we also detected lower proliferation, apoptosis, migration, and invasion in STK25-deficient HepG2 cells cultured without oleate supplementation; however, the effect of STK25 silencing was less pronounced in cells maintained under basal conditions (Figure 9*A–D*). Interestingly, reciprocal to STK25 knockdown, the overexpression of STK25 in HepG2 cells resulted in aggravated migration and invasion compared with the control cells transfected with the empty vector (Figure 10).

We further explored the expression levels of the markers of epithelial–mesenchymal transition (EMT) in HepG2 cells transfected with *STK25* siRNA vs nontargeting control siRNA. We found that STK25 knockdown markedly reduced the levels of mesenchymal markers fibronectin and N-cadherin and, reciprocally, enhanced the abundance of epithelial marker E-cadherin (Figures 8*E* and 9*B*). Furthermore, the silencing of STK25 decreased the transcript levels of *Slug* and *Zeb1* (Figure 8*H* and 9*E*), which are the critical transcriptional factors regulating EMT of cancer cells.^47^

Impaired liver autophagy caused by hepatitis C virus and hepatitis B virus infection and lipid toxicity is closely related to the pathogenesis of HCC.^18^ To this end, we found that the silencing of STK25 in HepG2 cells increased the ratio of LC3-I to LC3-II approximately 2-fold, which is an indicator of enhanced autophagic flux (Figure 8*I*).

In all, these data indicate that STK25 antagonism suppresses the metastatic ability of HCC cells by inhibiting EMT and regulating autophagy.

## Discussion

This study describes sterile 20-type kinase STK25 as a crucial promoter of hepatocellular carcinogenesis in the context of NASH. Using the DEN- and CDAA-induced models of HCC, we found that the livers from *Stk25* knockout mice showed markedly lower tumor burden compared with wild-type littermates, which was accompanied by reduced apoptosis and compensatory cell proliferation, as well as suppressed neoangiogenesis. Interestingly, in our study, the expression of AFP, YAP, GRP78, and EpCAM was attenuated in the livers from *Stk25*^*-/-*^ mice, supporting the importance of STK25 not only in the development of HCC but also in tumor progression.

Although mortality associated with most cancer types has decreased steadily over the past decades, both the incidence rate and deaths resulting from HCC have increased sharply.^48^ Several anticancer drugs are available for HCC therapy today (regorafenib, sorafenib, and nivolumab have received regulatory approval both by European Medicines Agency and the Food and Drug Administration); however, the overall 5-year survival rate remains 30%–40%, and the recurrence rate is approximately 80% in patients with advanced HCC.^49^ Importantly, the development of efficient anti-HCC therapies has been hampered by the poor understanding of molecular pathogenesis of HCC, and NASH-driven HCC in specific. Interestingly, several recent studies have emphasized the impact of lipid metabolism in HCC genesis with intrahepatocellular lipid accumulation inducing oxidative and ER stress as well as inflammation, which subsequently accelerate tumorigenesis.^19^^,^^33^^,^^39^ In this study, in both the DEN- and CDAA-induced models of HCC, the genetic ablation of STK25 protected the mice against hepatic lipid accumulation, which was accompanied by lower generation of ROS, suppressed ER stress, as well as reduced infiltration of inflammatory cells in the liver. Thus, STK25 is likely controlling HCC initiation and progression by altering the susceptibility to hepatic lipotoxicity.

We further show that depletion of STK25 suppresses hepatocellular tumor growth in mice through reduced apoptosis and lowered compensatory proliferation by a mechanism that involves the inactivation of STAT3, ERK1/2, and p38 signaling—the key pathways also implicated in human HCC.^41^^,^^42^^,^^46^ Because the subcellular localization of STK25 is strictly confined to intrahepatocellular lipid droplets both in mouse and human liver cells,^12^^,^^13^^,^^17^ it is unlikely to directly phosphorylate these targets. Thus, the exact mechanisms by which STK25 alters STAT3, ERK1/2, and p38 activation remain elusive.

EMT has been widely recognized as a critical mechanism of cancer metastasis, an important reason for the poor prognosis of HCC patients.^50^ To this end, it is significant that we detected suppressed mobility and invasiveness, as well as repressed expression of key molecular drivers of EMT, in STK25-deficient HepG2 cells, suggesting an additional mechanism underlying the lower HCC burden detected in *Stk25*^-/-^ mice in vivo.

A study by Lim et al^51^ recently identified STK25 as an activator of the Hippo signaling pathway by directly phosphorylating and activating LATS1/2. Hippo signaling normally acts to maintain tissue homeostasis and to suppress oncogenic co-activators YAP and TAZ. Consistent with this, STK25 deficiency was shown to lead to a moderate, but significant, increase in liver mass in mice at 6–12 months of age; however, liver carcinomas were not identified in any of the animals.^51^ In contrast, a recent report by Bae et al^52^ described STK25 as a suppressor of Hippo pathway activation by inhibiting mammalian sterile 20-like kinase (MST2). Thus, STK25 may have both positive and negative effects on this pathway, depending on the downstream signaling modality. Interestingly, messenger RNA expression analysis in hepatic tumors from more than 350 subjects also has identified STK25 as an unfavorable prognostic marker in human liver cancer because high expression of STK25 was associated with a lower 5-year survival rate compared with low expression.^53^^,^^54^ In line with these previous observations, in this study we found reduced hepatocyte proliferation and lower liver YAP abundance in HCC tumors from *Stk25* knockout mice.

Although the genetic deletion of STK25 suppressed hepatocellular carcinogenesis, it did not completely prevent the formation of HCC in mice. Notably, STK25 belongs to the GCKIII subfamily of sterile 20-type kinases together with 2 closely related kinases MST3 (also known as STK24) and MST4, which all are associated with lipid droplets in the liver and have similar functions regulating hepatocyte lipotoxicity and metabolic stress response in the context of obesity.^12^, ^13^, ^14^^,^^17^^,^^55^, ^56^, ^57^ It therefore is possible that antagonizing the activity of all 3 of these GCKIII kinases may promote the anti-HCC effect further.

In summary, we provide evidence that abrogation of STK25 hinders the acceleration of HCC development in mice in the context of NASH. The antitumor effect seen in mice by antagonizing the STK25 signaling pathway may open up new avenues for NASH-induced HCC treatment in human beings.

## Materials and Methods

### Animal Experiments

*Stk25* knockout mice along with their wild-type littermates were generated and genotyped as reported earlier.^27^ The male mice were weaned at 3 weeks of age and housed 3–5 per cage in a temperature-controlled (21°C) facility with a 12-hour light/dark cycle and ad libitum access to chow and water.

To induce HCC, a single intraperitoneal injection with DEN (25 mg/kg, N0258; Sigma-Aldrich, St. Louis, MO) was performed in 14-day-old *Stk25*^-/-^ and wild-type mice. Starting from 4 weeks after the DEN injection, mice were subjected to high-fat diet (60 kcal% fat, D12492; Research Diets, New Brunswick, NJ) feeding until 36 weeks of age.

HCC also was induced in another cohort of *Stk25*^-/-^ and wild-type mice by CDAA diet (30 kcal% fat-corn oil and palm oil, A18092701; Research Diets) feeding from the age of 6 to 43 weeks. During the CDAA diet-feeding period, mice were injected once weekly with a low-dose (0.2 μL/g of body weight) of CCl_4_ (289116; Sigma-Aldrich) diluted (1:4 ratio) with corn oil (C8267; Sigma-Aldrich).^22^

At age 36 weeks (for the DEN model) and 43 weeks (for the CDAA diet model), mice were killed after withholding food for 4 hours. To investigate the cell proliferation, mice were injected intraperitoneally with BrdU (100 mg/kg, B5002; Sigma-Aldrich) 2 hours before death.^32^ Blood was collected through heart puncture. Liver tissue was weighed, and each lobe of liver was photographed. Whole (tumor-bearing) liver tissue was collected for histologic and immunofluorescence/immunohistochemistry analysis as described later. Liver tumors also were isolated, snap frozen in liquid nitrogen, and stored at -80°C for Western blot analysis of protein expression.

The mice used in the study received humane care described by the National Institutes of Health (Bethesda, MD) recommendations outlined in the *Guide for the Care and Use of Laboratory Animals*. All the in vivo experiments were performed according to the protocols approved by the local Ethics Committee for Animal Studies at the Administrative Court of Appeals in Gothenburg, Sweden, following appropriate guidelines.

### Liver Tumor Parameters

The photographs of liver lobes were used to measure the following tumor parameters: maximal tumor volume (the volume of the largest visible HCC tumor found in each liver), total tumor volume (the sum of the volumes of all visible HCC tumors in each liver), and total tumor number.

### Histologic and Immunofluorescence Analysis of Liver Sections

Liver tissue was fixed in 4% (vol/vol) phosphate-buffered formaldehyde (Histolab Products, Gothenburg, Sweden), embedded in paraffin, and sectioned. Paraffin sections were stained with H&E (Histolab Products) for morphologic analysis, with Picrosirius Red (Histolab Products) for examination of the degree of fibrosis, or with dihydroethidium (Life Technologies, Grand Island, NY) to measure superoxide radical formation. Apoptotic cells were identified by using the horseradish-peroxidase–3,3′-diaminobenzidine tetra hydrochloride TUNEL Assay Kit (Abcam, Cambridge, UK).

Liver samples also were embedded in optimal cutting temperature mounting medium (Histolab Products) and frozen in liquid nitrogen, followed by cryosectioning. Cryosections were stained with Oil Red O (Sigma-Aldrich) to access neutral lipids or MitoTracker Red (Thermo Fisher Scientific, Waltham, MA) for detection of respiring mitochondria. The labeled area was quantified in 6–8 randomly selected microscopic fields (×200; distributed over 3 nonconsecutive liver sections) per mouse using ImageJ software (version 1.47; National Institutes of Health).

For immunofluorescence or immunohistochemistry analysis, paraffin sections or cryosections were incubated with primary antibodies, followed by incubation with fluorescent dye–conjugated or biotinylated secondary antibodies (see Table 1 for antibody information). The total area labeled was quantified in 8 randomly selected microscopic fields (×100 or ×200, distributed over 3 nonconsecutive liver sections) per mouse using ImageJ software.

**Table 1:** List of Antibodies Used for Western Blot and Immunofluorescence Analysis

Type	Antibody name and catalog number	Working dilution	Company
Primary	Anti-STK25 (25821-1-AP)	1:750	Proteintech (Chicago, IL)
antibody	Anti-AFP (ab46799)	1:200	Abcam
	Anti-YAP (ab205270)	1:500	Abcam
	Anti-GRP78 (sc-166490)	1:250	Santa Cruz Biotechnology (Santa Cruz, CA)
	Anti-EpCAM (ab213500)	1:100	Abcam
	Anti–cleaved CASP3 (ab49822)	1:500	Abcam
	Anti-BrdU (ab6326)	1:100	Abcam
	Anti-Ki67 (14-5698-82)	1:200	Invitrogen (Waltham, MA)
	Anti-PCNA (MA5-11358)	1:200	Invitrogen
	Anti–CK-19 (ab15463)	1:100	Abcam
	Anti-CD31 (558736)	1:100	BD Biosciences (San Jose, CA)
	Anti-F4/80 (MCA497GA)	1:250	Bio-Rad
	Anti-Gr1 (Ly6C) (ab15627)	1:300	Abcam
	Anti–collagen IV (ab6586)	1:200	Abcam
	Anti-fibronectin (F3648)	1:300	Sigma-Aldrich
	Anti-αSMA (ab5694)	1:200	Abcam
	Anti–8-oxoguanine (ab62623)	1:500	Abcam
	Anti-γH2Ax (ab81299)	1:250	Abcam
	Anti–4-HNE (ab46545)	1:500	Abcam
	Anti-E06 (330001S)	1:100	Avanti Polar Lipids, Inc (Alabaster, AL)
	Anti-KDEL (ab176333)	1:500	Abcam
	Anti-CHOP (MA1-250)	1:200	Invitrogen
	Anti-ERK1/2 (#9102)	1:1000	Cell Signaling Technology (Boston, MA)
	Anti–p-ERK1/2 (#9101)	1:1000	Cell Signaling Technology
	Anti-JNK (#9252)	1:1000	Cell Signaling Technology
	Anti–p-JNK (#4668)	1:500	Cell Signaling Technology
	Anti-p38 (#9212)	1:1000	Cell Signaling Technology
	Anti–p-p38 (#9211)	1:500	Cell Signaling Technology
	Anti-STAT3 (#4904)	1:1000	Cell Signaling Technology
	Anti–p-STAT3 (#9131)	1:500	Cell Signaling Technology
	Anti-actin (#A2920)	1:500	Santa Cruz Biotechnology
	Anti-GAPDH (sc-47724)	1:1000	Santa Cruz Biotechnology
	Anti–N-cadherin (33-3900)	1:500	Invitrogen
	Anti–E-cadherin (14-3249-82)	1:200	Invitrogen
	Anti-LC3B (#2775)	1:1000	Cell Signaling Technology
Secondary antibody	Alexa Fluor–488–labeled anti-mouse IgG (A21202)	1:500	Invitrogen
	Alexa Fluor–488–labeled anti-rabbit IgG (A11008)	1:500	Invitrogen
	Alexa Fluor–594–labeled anti-mouse IgG (A11005)	1:500	Invitrogen
	Alexa Fluor–594–labeled anti-rabbit IgG (A21207)	1:500	Invitrogen
	Alexa Fluor–594–labeled anti-rat IgG (A11007)	1:500	Invitrogen
	Anti-rat (biotin) (ab6733)	1:300	Abcam
	Anti-rabbit IgG (#7074)	1:1000	Cell Signaling Technology
	Anti-mouse IgG (#7076)	1:1000	Cell Signaling Technology

4-HNE, 4-hydroxynonenal; αSMA, α smooth muscle actin; CHOP, C/EBP homologous protein; CK-19, cytokeratin-19; GAPDH, glyceraldehyde-3-phosphate dehydrogenase.

### Cell Culture, RNA Interference, and Transient Overexpression

HepG2 cells (hepatocellular carcinoma, human; American Type Culture Collection, Manassas, VA) were maintained in Dulbecco's modified Eagle medium (GlutaMAX supplemented; Gibco, Paisley, UK) supplemented with 10% (vol/vol) fetal bovine serum and 1% (vol/vol) penicillin/streptomycin (Gibco). Cells were shown to be free of mycoplasma infection by the MycoAlert Mycoplasma Detection Kit (Lonza, Basel, Switzerland). Cells were transfected with *STK25* siRNA (s20570; Ambion, Austin, TX) or scrambled siRNA (SIC001; Sigma-Aldrich) using Lipofectamine RNAiMax and Opti-MEM (Thermo Fisher Scientific). Cells also were transfected with human *FLAG-STK25* (GeneCopoeia, Rockville, MD) or an empty control plasmid using Lipofectamine 2000 and Opti-MEM (Thermo Fisher Scientific). In most experiments described later, transfected cells were exposed to 100 μmol/L oleic acid (Sigma-Aldrich) for 48 hours, which efficiently induces steatosis in vitro.

Cells were fixed in 4% (vol/vol) phosphate-buffered formaldehyde (Histolab Products) and then processed for immunofluorescence with anti-Ki67, anti-PCNA, anti-cleaved CASP3, anti-fibronectin, anti–E-cadherin, or anti–N-cadherin antibodies (see Table 1 for antibody information). The labeled area was quantified in 6–10 randomly selected microscopic fields (×200) per well using ImageJ software. Proliferation was measured using the BrdU-Labeling Assay Kit (Amersham Cell Proliferation Biotrak enzyme-linked immunosorbent assay System; GE Healthcare, Chicago, IL). To assess migration, cells were added to the upper chambers of the Transwells with 8-μm pore size (Polycarbonate Cell Culture Inserts in Multi-Well Plates; Costar, Kennebunk, ME) and medium with 10% fetal bovine serum was added in the bottom chambers for a chemotactic gradient. After 24 hours, cells on the upper surface were removed using cotton swabs, and the cells on the bottom side of the membranes were fixed and stained with 0.1% crystal violet (Sigma-Aldrich). To determine the invasive capacity of cells, the Transwells were coated with Matrigel matrix (Corning, Bedford, MA) before the experiment.

### Quantitative Real-Time Polymerase Chain Reaction

RNA was isolated from HepG2 cells with the EZNA Total RNA Kit (Omega Bio-Tek, Norcross, GA) according to the manufacturer’s recommendations. Complementary DNA was synthesized using the High-Capacity Complementary DNA Reverse Transcription Kit (Applied Biosystems, Foster City, CA). Relative quantification was performed with the CFX Connect Real-Time System (Bio-Rad, Hercules, CA). Relative quantities of target transcripts were calculated after normalization of the data to the endogenous control, 18S ribosomal RNA (Applied Biosystems).

### Western Blot

Western blot was performed as previously described^58^ (see Table 1 for antibody information).

### Statistical Analysis

Statistical significance between the groups was evaluated using the 2-sample Student *t* test with *P* < .05 considered statistically significant. All statistical analyses were performed using SPSS statistics (v24; IBM Corporation, Armonk, NY).
