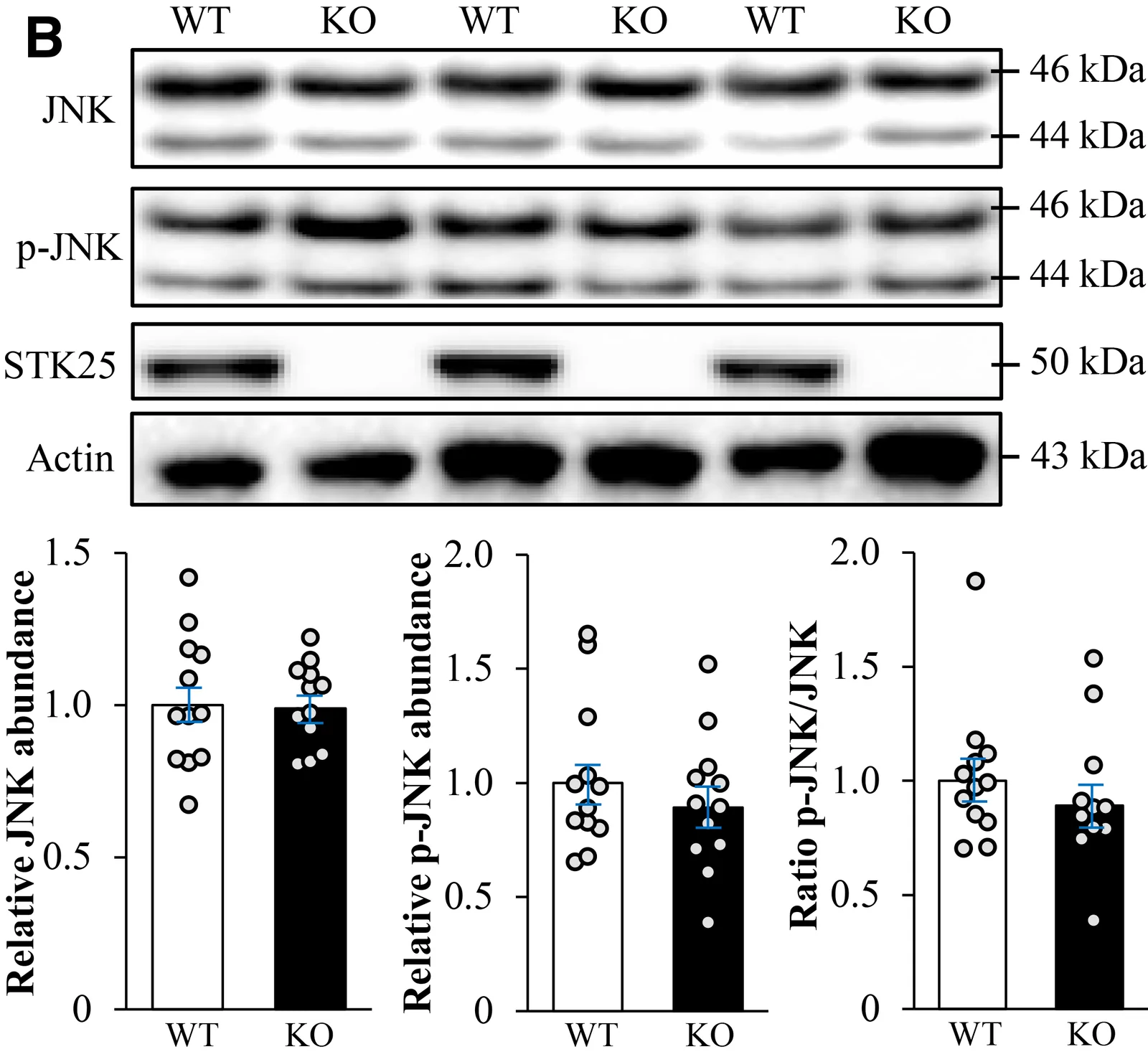# Correction

**DOI:** 10.1016/j.jcmgh.2026.101898

**Published:** 2026-10-31

Kurhe Y, Caputo M, Cansby E, et al. Antagonizing STK25 Signaling Suppresses the Development of Hepatocellular Carcinoma Through Targeting Metabolic, Inflammatory, and Pro-Oncogenic Pathways. Cell Mol Gastroenterol Hepatol 2022;13:405-423.

In the above article, an error occurred during the preparation of Figure 7*B*, which contained a mismatched representative actin image. The authors apologize for this error. The corrected figure is shown below. This correction does not affect the quantification of target protein levels or the conclusions of the analysis but is important to ensure accurate presentation of the data.

**Figure 7B:**